# Is debridement beneficial for focal cartilage defects of the knee: data from the German Cartilage Registry (KnorpelRegister DGOU)

**DOI:** 10.1007/s00402-020-03338-1

**Published:** 2020-01-22

**Authors:** Manuel Weißenberger, Tizian Heinz, Sebastian P. Boelch, Philipp Niemeyer, Maximilian Rudert, Thomas Barthel, Stephan Reppenhagen

**Affiliations:** 1 Department of Orthopaedic Surgery, University of Wuerzburg, Koenig-Ludwig-Haus, Brettreichstr. 11, 97074 Wuerzburg, Germany; 2 OCM Clinic, Steinerstr. 6, 81369 Munich, Germany; 3 Department of Orthopaedics and Trauma Surgery, Freiburg University Hospital, Hugstetter Str. 55, 79106 Freiburg im Breisgau, Germany

**Keywords:** Articular cartilage, Focal cartilage defects, Osteoarthritis, Knee surgery, Arthroscopic debridement, Meniscal surgery, KOOS, NRS

## Abstract

**Introduction:**

Focal cartilage defects of the knee are often treated with arthroscopic debridement. Existing literature discussing the benefit of debridement for small articular cartilage lesions is scarce, especially if the debridement was not part of a combined operative cartilage procedure including meniscal and ligament repair. The purpose of this study was to examine the patients´ benefit after arthroscopic debridement for the treatment of isolated focal chondral defects with or without partial meniscus resection.

**Materials and methods:**

Baseline (preoperative data) and 12-month follow-up of the five Knee Osteoarthritis Outcome Score (KOOS) subscores and the Numeric Rating Scale (NRS) for pain were analyzed in 126 patients undergoing debridement for focal chondral defects of the knee from the German Cartilage Registry. Sub-analysis for patients receiving isolated debridement and debridement with concomitant partial resection of meniscal pathologies was performed. Thus, four subgroups were created according to the treated defect size and presence of meniscal pathologies: “debridement-only < 2 cm^2^”, “debridement-only > 2 cm^2^”, “debridement and partial meniscus resection < 2 cm^2^” and “debridement and partial meniscus resection > 2 cm^2^”.

**Results:**

KOOS-subscores showed a significant increase from baseline to follow-up evaluation (*p* = 0.017–0.037) within the 126 patients. Sub-analysis showed significant improvement of all five KOOS-subscores in all three subgroups, except for the “debridement and partial meniscus resection > 2 cm^2^”—group: in this group the KOOS subscores symptoms and sports showed no significant improvement. The NRS scores revealed no significant changes from baseline to 12-month follow-up within the four subgroups.

**Conclusion:**

An overall benefit of arthroscopic debridement for focal cartilage lesions of the knee could be conducted. Isolated cartilage defects seem to benefit from debridement irrespectively of size. In patients with large cartilage defects (> 2 cm^2^) and concurrent meniscal pathology expectation to improvement should be humbled. Effective reduction of pain by arthroscopic debridement remains unclear.

## Introduction

Focal cartilage defects of the knee are difficult to treat. Concomitantly, they are often associated with pain, swelling, instability, popping symptoms and impairment in daily life. These defects are considered to have limited self-healing characteristics and thus a propensity for progression of an early onset osteoarthritis [1–4]. Notably, in more than 50% of all knee arthroscopies performed, articular cartilage defects can be detected, though often being asymptomatic [5–7].

In the United States palliative (e.g. lavage chondroplasty) and restorative (autologous chondrocyte implantation, osteochondral transplantation) techniques are predominantly used for cartilage repair. In this regard, approximately 300,000 patients underwent arthroscopic surgery due to focal cartilage defects of the knee in 2010. Of these, roughly 220,000 patients were treated with arthroscopic debridement; however, the proportion of these patients undergoing concomitant surgery is unclear [8]. Due to the high prevalence of arthroscopic debridement for focal cartilage lesions of the knee joint, knowledge on the overall utility of this treatment method is of utmost importance. In particular, the question remains whether this operative strategy is a suitable treatment option for focal articular cartilage defects. Presently, most studies focus on a heterogeneous and frequently ill-defined patient population. Furthermore, data provided especially regarding the clinical outcome of this treatment option is inconclusive [9–11].

Today, there is a scientific consensus that debridement of large osteoarthritis like lesions of the articular cartilage of the knee should be avoided. Yet, no definite treatment recommendation can be made for debridement of focal cartilage defects, especially those ranging from grade II–III according to the International Cartilage Repair Society (ICRS)-classification system [12]. Guidelines and therapy algorithms for higher grade (ICRS-grade IIIc–IV) defects have already been established by the “Working Group on Tissue Regeneration” of the German Society for Orthopaedics and Trauma (DGOU). Likewise, conservative therapy options for low-grade cartilage defects (ICRS-grade I) have found broad acceptance [12, 13]. In a survey performed in 2009 by the German Society for Arthroscopy and Joint Surgery (AGA), 246 surgeons were asked about their operative procedure habits regarding focal cartilage defects. Interestingly, 53% stated to perform arthroscopic debridement for symptomatic ICRS-grade I–II defects [14]. However, the overall utility remains debatable.

For this reason, the purpose of this study was to analyze current clinical multicenter data of patients undergoing arthroscopic debridement for focal cartilage defects. These data were part of the German Cartilage Registry [KnorpelRegister Deutsche Gesellschaft für Orthopädie und Unfallchirurgie (DGOU)]. Particularly, the aim was to provide a deeper insight into this routinely performed surgical procedures for cartilage repair in Germany. The main hypothesis of this multicenter analysis was that patients with focal cartilage defects of the knee joint benefit from debridement in the short-term follow-up over 12 months.

## Materials and methods

### German Cartilage Registry (KnorpelRegister DGOU)

The German Cartilage Registry (KnorpelRegister DGOU) is a nationwide and multicenter organized registry for patients undergoing cartilage repair surgery. The aim is to provide a structural follow-up of clinical outcome data as well as a deep insight of the real-life treatment patterns for cartilage defects [15]. Since the introduction of the registry in October 2013, a total of 3919 patients assigned for cartilage treatment of the knee joint were registered through 141 centers in Germany, Austria and Switzerland by the end of 2017. The registry is conducted in accordance with the Declaration of Helsinki and registered at germanctr.de (DRKS00005617). Patient-specific characteristics such as age, sex, weight [body mass index (BMI)] and intraoperative defect specific parameters (e.g. defect size, defect localization, ICRS-grade of the defect, operative procedure applied) are entered by the physician. Meanwhile, the patient is asked to fill in validated questionnaires such as the Knee Osteoarthritis Outcome Score (KOOS) and the Numeric Rating Scale (NRS) for pain to assess clinical symptoms and functional outcome. In every case, informed and signed consent had to be given by the patients to be eligible for the cartilage registry. Links to the questionnaires are sent out automatically to the patients’ email-addresses at specific time points and are only accessible four weeks after the link was sent out (preoperatively as well as 6, 12, 24, 36, 60 and 120 months after intervention).

### Data acquisition and analysis

**Fig. 1:**
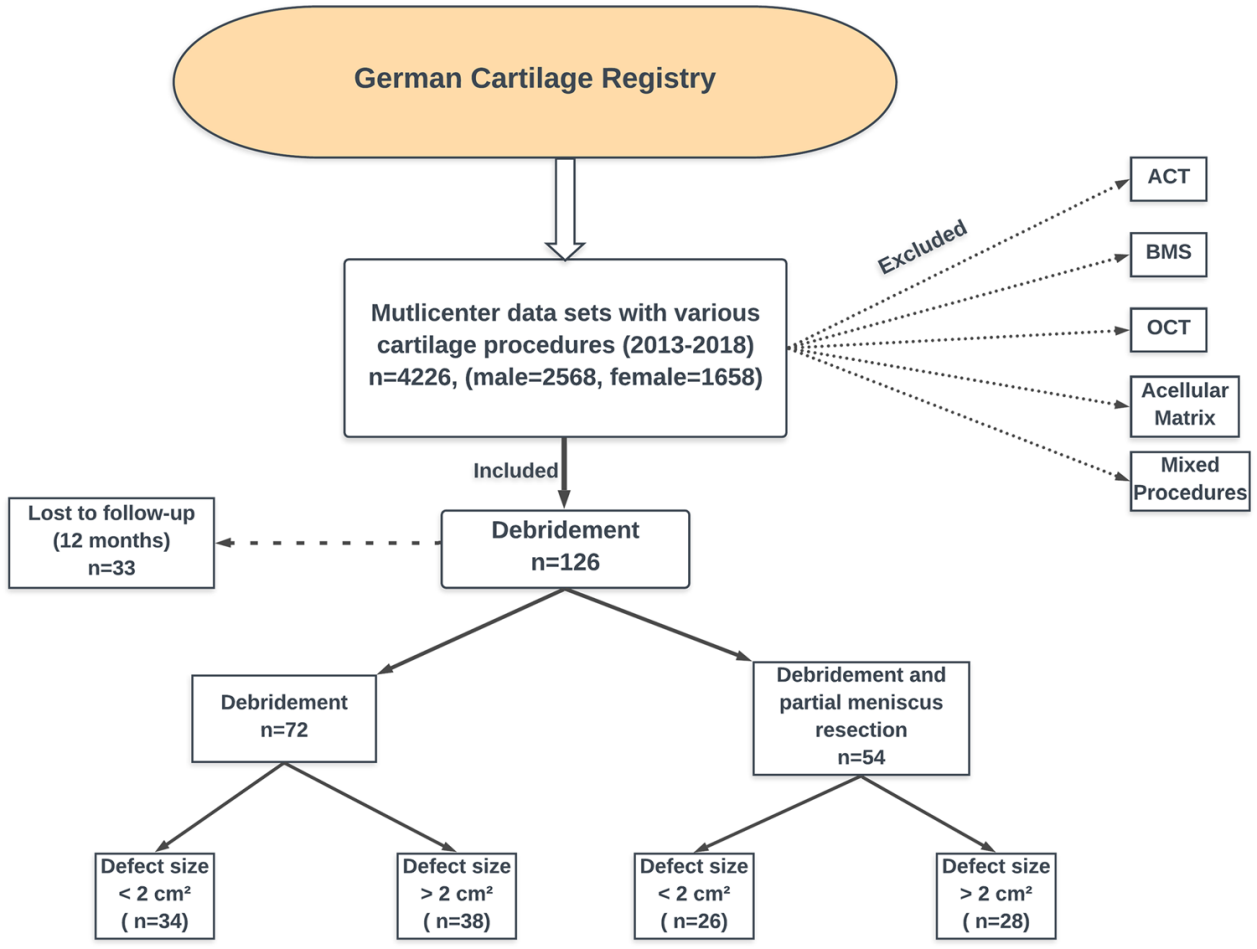
Flowchart of the data acquisition process. Multicenter data from study centers across Germany were included over a period of about five years

A multicenter dataset was obtained from the German Cartilage Registry (DGOU) encompassing a total of 4226 patients undergoing surgery for various cartilage conditions (date of database inquiry: February 2019). After carefully reviewing the dataset a total of 126 patients treated by debridement or debridement with concomitant partial meniscus resection, meeting the undermentioned criterions, were found eligible for the study (Fig. 1). Inclusion criterions were a focal cartilage mono-defect of the knee treated by arthroscopic debridement and absence of any concomitant pathologies of the joint other than the focal cartilage defect and the eventually accompanying meniscal lesion. This also means that any degree of osteoarthritis of the knee was excluded. Osteoarthritis was defined as multiple and broad cartilage defects extending to the whole joint area resembling a degenerative joint wear. Cartilage defects were graded according to the ICRS classification during arthroscopy. Only cartilage mono-defects were treated by debridement. Furthermore, cartilage defects were recorded in the German Cartilage Registry with defect localization and size. Different meniscal tear patterns could not be differentiated in the German Cartilage Registry.

Patients had to be at least 18 years of age and in possession of a valid email-address for the registration process.

The KOOS as a patient-reported outcome score (PRO) was evaluated and scored according to existent scoring recommendations [16]. KOOS and NRS belonged to multicenter datasets of the German Cartilage Registry. Sample means, standard deviation and 95% confidence intervals (CI) were calculated for PROs at baseline and 12 months after surgical intervention. Patients were grouped either into “treated with debridement of the cartilage defect only” or into “patients undergoing concomitant partial resection of the meniscus”. Furthermore, each of these groups was divided into two further subgroups based on the defect size of the cartilage. As regards, lesions < 2 cm^2^ were considered small, in contrast lesions > 2 cm^2^ were considered intermediate to big. Means of the clinical outcome measures of the KOOS and NRS were calculated for each subgroup at baseline as well as at 12-month follow-up.

### Statistics

Data evaluation and statistical analysis were performed using IBM SPSS Statistics version 25 (IBM Corp., Armonk, NY, USA) and XLSTAT (XLSTAT 2018: Data Analysis and Statistical Solution for Microsoft Excel. Addinsoft, Paris, France). Descriptive data are presented as mean ± standard deviation or percentage of the total. Normal distribution of the data was evaluated with the Kolmogorov–Smirnov test. Paired samples were compared with the paired samples *t* test in case of normal distribution and with the Wilcoxon test otherwise. Unpaired samples were tested for significant difference with the independent *t* test in case of normal distribution and with the Mann–Whitney *U* test otherwise. An ANOVA was performed to identify significant differences between the four subgroups. Post-hoc analysis was done with the Tukey test in case of homogeneity of variances and with the Tamhane test otherwise. Significance was assumed if *p* was < 0.05. All statistics were conducted with SPSS Version 25 (IBM).

Regarding the amount of missing data, about 27% of values were missing at baseline, reaching about 35% at 12-month follow-up.

## Results

### Patient and defect characteristics

The debridement-only-group, as well as the debridement-group that underwent concomitant partial meniscus resection was further sub-grouped into two groups according to its defect size. Characterization by baseline demographics and surgical findings was done for each group (Tables 1, 2). Group sizes ranged 26–38 patients. In all four groups the medial femoral condyle was the most often located defect area (52.9–61.5%, 70 patients in total), followed by retropatellar cartilage defects (10.7–23.7%, 25 patients in total). Patients with meniscal pathologies were slightly older compared to patients with focal cartilage lesions only (50.7 years vs. 45.3 years). Patients with large cartilage defects > 2 cm^2^ had also a slightly higher mean age compared to patients with small cartilage lesions < 2 cm^2^ (55.3 years vs. 44.8 years). Cartilage defects were characterized by the ICRS classification: Defect lesions ICRS IIIa–c were predominantly present (47.1–57.7%, 68 patients in total). Cartilage defects ICRS I and II were found in 30 patients (23.8%). BMI ranged 26.5–28.8 kg/m^2^. There were slightly more male patients in each subgroup. During the follow-up of the study a total of 33 patients were lost to follow-up.

**Table 1:** Multicenter data

Debridement only, defect size < 2 cm^2^		Debridement only, defect size > 2 cm^2^	
Variable	Mean ± SD or fraction (%)	Variable	Mean ± SD or fraction (%)
Age, years	41.4 ± 15.8	Age, years	48.7 ± 13.2
BMI	26.5 ± 5.9	BMI	28.8 ± 4.4
Gender, male/female, *n*	16/18	Gender, male/female, *n*	20/18
Lesion size, cm^2^	1.1 ± 0.5	Lesion size, cm^2^	4.0 ± 1.4
Localization of defect (*N *= 34)		Localization of defect (*N* = 37)	
Medial FC	18 (52.9)	Medial FC	21 (55.3)
Lateral FC	3 (8.8)	Lateral FC	1 (2.6)
Retropatellar	8 (23.5)	Retropatellar	9 (23.7)
Tibial plateau	2 (5.8)	Trochlea	5 (13.2)
Trochlea	3 (8.8)	Tibial plateau	1 (2.6)
ICRS-grade (*N *= 34)		ICRS-grade (*N *= 38)	
2	9 (26.5)	2	8 (21.0)
3a–c	16 (47.1)	3a–c	21 (55.3)
4a/4b	9 (26.5)	4a/b	9 (23.7)

Patient demographics and surgical characteristics for the debridement-only-group with a defect size < 2 cm^2^ (left column) and a defect size > 2 cm^2^ (right column)

*BMI* body mass index, *ICRS* International Cartilage Repair Society, *FC* femoral condyle

**Table 2:** Multicenter data

Debridement and partial meniscus resection, defect size < 2 cm^2^		Debridement and partial meniscus resection, defect size > 2 cm^2^	
Variable	Mean ± SD or fraction (%)	Variable	Mean ± SD or fraction (%)
Age, years	49.9 ± 11.0	Age, years	52.7 ± 9.1
BMI	28.2 ± 4.3	BMI	27.6 ± 3.9
Gender, male/female, *n*	18/10	Gender, male/female, *n*	17/9
Lesion size, cm^2^	1.1 ± 0.4	Lesion size, cm^2^	3.6 ± 1.7
Localization of defect (*N *= 27)		Localization of defect (*N *= 26)	
Medial FC	15 (53.6)	Medial FC	16 (61.5)
Lateral FC	3 (10.7)	Lateral FC	3 (11.5)
Trochlea	3 (10.7)	Retropatellar	4 (15.4)
Tibial Plateau	2 (7.1)	Trochlea	3 (11.5)
Retropatellar	4 (14.3)		
ICRS grade (*N *= 28)		ICRS grade (*N *= 9)	
1	2 (7.1)	1	1 (3.8)
2	7 (25.0)	2	3 (11.5)
3a–c	16 (57.1)	3a–c	15 (57.7)
4a/b	3 (10.7)	4a/b	7 (26.9)

Patient demographics and surgical characteristics for the debridement with concomitant partial meniscus resection-group with a defect size < 2 cm^2^ (left column) and a defect size > 2 cm^2^ (right column)

*BMI* body mass index, *ICRS* International Cartilage Repair Society, *FC* femoral condyle

### Clinical outcome: KOOS, NRS

**Fig. 2:**
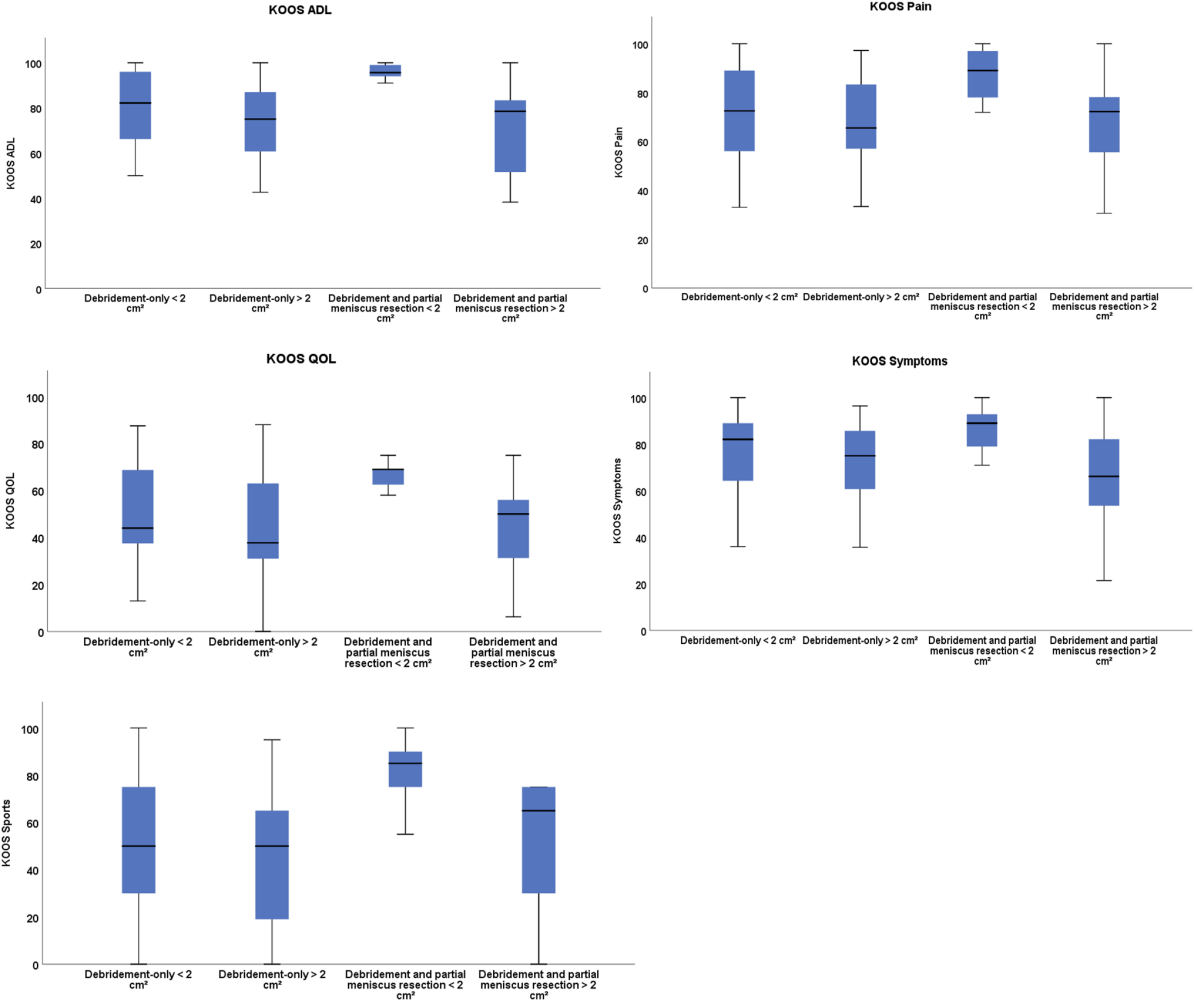
KOOS-subscale scores at 12-months follow-up for all debridement subgroups

All four debridement-subgroups improved from operative intervention by debridement. The KOOS-subscores showed no significant differences between the “debridement-only”-subgroups (Fig. 2). For the “debridement and partial meniscus resection”-subgroups significant differences in the KOOS-subscores were evident at 12-month follow-up (Table 3). When meniscal repair was applied, small defect sizes were associated with significantly higher outcome values (*p *= 0.00–0.04).

**Table 3:** Mean KOOS-Subscores at 12-months follow-up for the debridement with partial meniscus resection subgroups

Mean KOOS-subscore	Debridement and partial meniscus resection, defect size < 2 cm^2^	Debridement and partial meniscus resection, defect size > 2 cm^2^
KOOS-ADL 12 months (mean ± SD)	94.2 ± 6.5*^1^	73.0 ± 19.6*^1^
KOOS pain 12 months (mean ± SD)	88.7 ± 9.4*^2^	68.6 ± 19.1*^2^
KOOS QOL 12 months (mean ± SD)	66.0 ± 13.1*^3^	45.5 ± 23.0*^3^
KOOS symptoms 12 months (mean ± SD)	82.5 ± 15.7	66.6 ± 19.5
KOOS sports 12 months (mean ± SD)	80.8 ± 15.4*^4^	53.0 ± 26.2*^4^

Significant differences between the two groups in the mean KOOS-subscores are marked by asterisk

*P* values for statistically significant differences in KOOS-subscale scores between the debridement subgroups: (*1) *p *= 0.003, (*2) *p *= 0.005, (*3) *p *= 0.023, (*4) *p *= 0.04

Regarding the changes of the KOOS-subscores within a debridement-subgroup, improvement in all four subgroups during 12-month follow-up was observed. However, the degree of improvement was lower in the “debridement and partial meniscus resection > 2 cm^2^”-group (Table 4) (*p* = 0.00–0.04).

**Table 4:** Mean Difference within a KOOS-subscale from baseline to 12-months follow-up within a debridement subgroup

Change of KOOS (baseline to 12 months)	Debridement only, defect size < 2 cm^2^	Debridement only, defect size > 2 cm^2^	Debridement and partial meniscus resection, defect size < 2 cm^2^	Debridement and partial meniscus resection, defect size > 2 cm^2^
KOOS-ADL 12 months vs. PRE (mean ± SD)	12.4 ± 22.0*^1^	14.1 ± 24.7*^6^	27.6 ± 26.3*^11^	16.6 ± 23.1*^16^
KOOS pain 12 months vs. PRE (mean ± SD)	13.6 ± 27.0*^2^	18.8 ± 25.0*^7^	31.4 ± 24.4*^12^	13.4 ± 16.7*^17^
KOOS QOL 12 months vs. PRE (mean ± SD)	49.2 ± 20.4*^3^	19.4 ± 26.5*^8^	26.3 ± 24.0*^13^	16.4 ± 21.5*^18^
KOOS symptoms 12 months vs. PRE (mean ± SD)	8.0 ± 16.4*^4^	12.1 ± 21.9*^9^	21.6 ± 18.4*^14^	8.9 ± 20.6
KOOS sports 12 months vs. PRE (mean ± SD)	22.7 ± 34.4*^5^	30.4 ± 29.4*^10^	44.1 ± 32.6*^15^	17.2 ± 23.7

Significant increases within a KOOS-subscale are marked by asterisk. *p* values for significances are sorted by number and listed below

*P* values for statistically significant increase in a KOOS-subscale score within a debridement subgroup: (*1) *p *= 0.017, (*2) *p *= 0.031, (*3) *p *= 0.014, (*4) *p *= 0.037, (*5) *p *= 0.007, (*6) *p *= 0.014, (*7) *p *= 0.002, (*8) *p *= 0.003, (*9) *p *= 0.017, (*10) *p *= 0.000, (*11) *p *= 0.001, (*12) *p *= 0.000, (*13) *p *= 0.001, (*14) *p *= 0.000, (*15) *p *= 0.000, (*16) *p *= 0.012, (*17) *p *= 0.006, (*18) *p *= 0.008

**Fig. 3:**
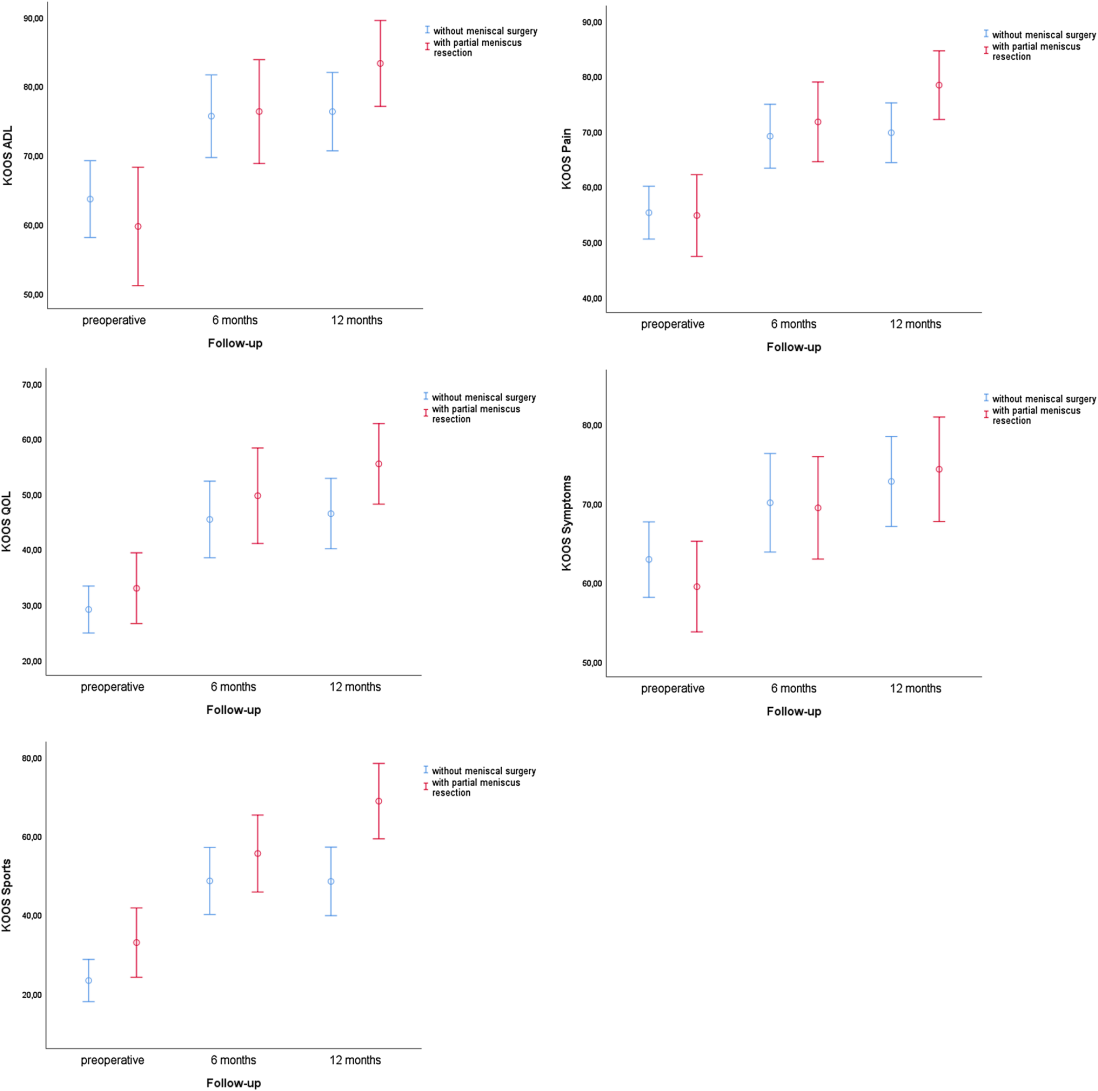
Mean KOOS-subscores with 95% confidential interval compared for single debridement and debridement with partial meniscus resection at specific follow-up points. No statistically significant difference for all KOOS-subscores could be shown

When omitting defect size separation for the “debridement-only”-group and for the “debridement and partial meniscus resection”-group, it could be shown that there were no significant differences in the KOOS-subscores regardless of whether partial meniscectomy was performed (Fig. 3).

**Fig. 4:**
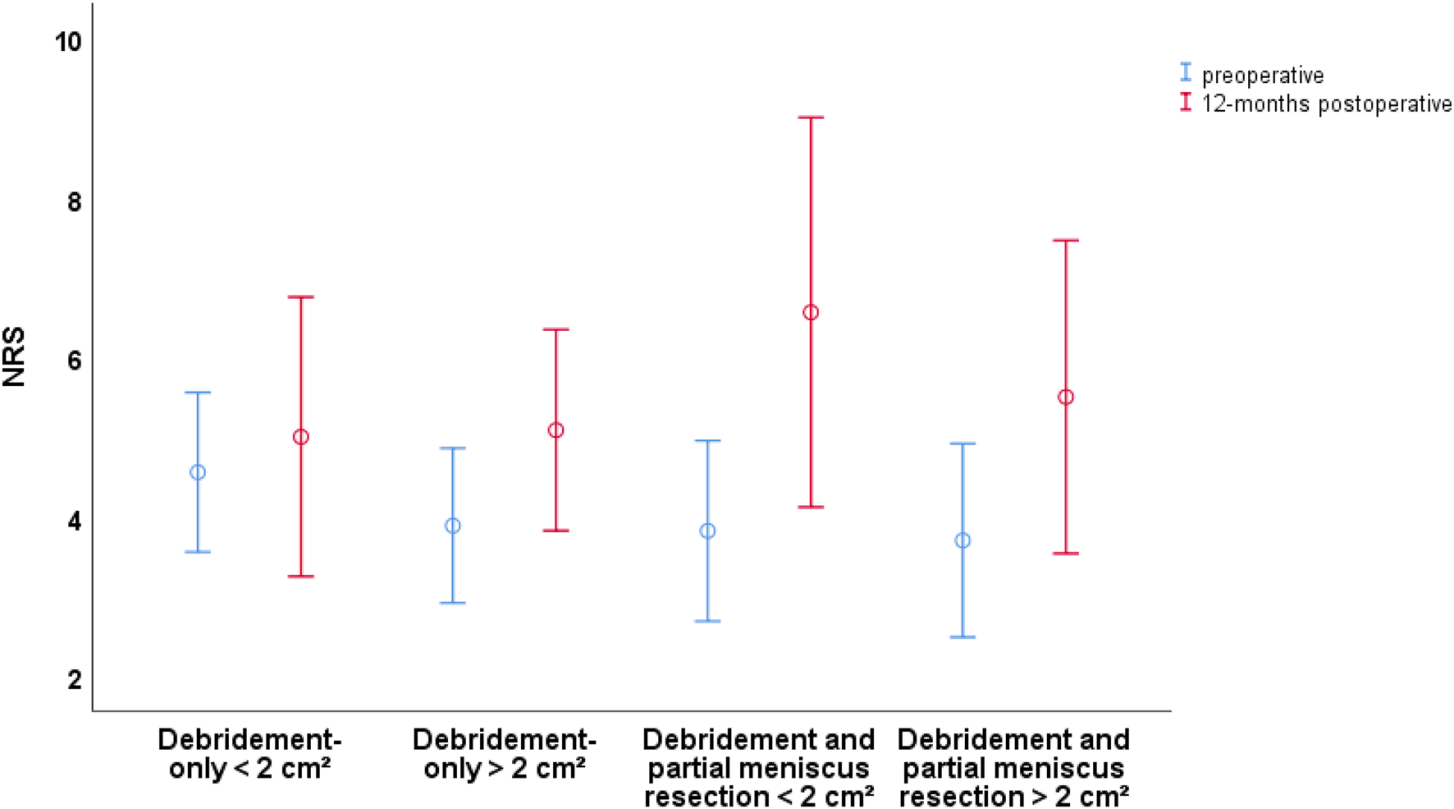
Mean value and 95% CI for the NRS of pain of all four subgroups at specific follow-up time points

Regarding the NRS of pain, no statistically significant change of the NRS could be found at baseline and 12-month follow-up between the subgroups (Fig. 4).

## Discussion

Articular cartilage defects of the knee are common findings afflicting up to 60% of patients that undergo knee arthroscopies [7, 17, 18]. Although chondroplasty is one of the most common arthroscopic procedures performed on the knee, only a sparse amount of literature reporting the clinical outcome for isolated cartilage defects after treatment with a single mechanical debridement is available [19]. Especially recent controversies regarding the use of debridement for osteoarthritis-like lesions seem to superimpose the possibility of cartilage debridement as a suitable and outcome improving therapy for small focal cartilage lesions. Currently, mechanical debridement has lost popularity because health insurances do not reimburse arthroscopic debridement, if any degree of osteoarthritis is present.

The major finding of this study was that patients with a focal cartilage defect without any meniscal pathology can have a functional improvement in the short-term follow-up. As far as known, in most cases where debridement for focal cartilage defects has been reported to be used, it has not been performed as a single procedure for cartilage repair but in addition to a number of surgical procedures. These include procedures such as meniscectomy and ligament repair [10, 12], making it difficult to determine the real influence of debridement on the total functional outcome score. However, most of these studies showed an overall improvement in knee function concordant with the results of this study [12].

Another important finding was the missing correlation of the KOOS-Pain score, showing an overall improvement in all subgroups, with the NRS score for pain that did not change significantly during the whole follow-up period of 12 months. This means that the benefit of effectively reducing the pain through arthroscopic debridement remains unclear. Therefore, further studies investigating the comparability and sensitivity of the KOOS Pain and NRS score for pain in a cartilage repair population would be desirable.

Of note, increasing defect sizes (> 2 cm^2^) are associated with minor clinical improvements as only three of five KOOS-subscales showed a significantly higher score at follow-up for the debridement and partial meniscus resection group. This result is consistent with the existing literature questioning the benefit of debridement for big osteoarthritis like lesions [20, 21]. Though newer studies suggest a benefit at least for patients with osteoarthritis and non-traumatic flap tears of the medial meniscus [22].

A recent study of Anderson et al. concluded that an isolated mechanical chondroplasty of focal cartilage lesions of the knee is beneficial to patients in absence of any concurrent pathology [19]. This seems to be consistent with the findings of this study. It could be shown that KOOS subscores differed not significantly for the debridement-only procedure compared to the debridement with partial meniscectomy. In a broader sense, data of this study tend to support the hypothesis that debridement for focal, partial thickness cartilage defects is at least as effective as combined with partial meniscectomy for meniscal repair.

Collectively, recent treatment recommendations of the “Working Group on Tissue Regeneration” of the German Society of Orthopaedics and Trauma justifying arthroscopic debridement for symptomatic focal, unstable chondral defects, can be supported [12].

Of course, it should be considered that measurement properties of the KOOS have been validated for various knee pathologies including chondral lesions and meniscal injuries, in case of the KOOS even for mild osteoarthritis [23–30]. Furthermore, Tanner et al. concluded that the KOOS contains the most items of general knee instruments important to patients [30]. However, one has to be aware that the KOOS is still a subjective patient reported outcome score lacking objective correlation, making it easy to overestimate and misinterpret findings.

There are several limitations accompanying this study design that are worth mentioning. Firstly, the presented findings need to be considered as short-term results with follow-up periods of 6 and 12 months. Hence, further studies are necessary to provide long-term results showing the development of the clinical outcome even several years after intervention. Another limitation of this study is that it comprises a relative small amount of patients treated by debridement and the use of subjective PROs without objective clinical and radiological examination in the follow-up.

Generally, high-quality registries like the German Cartilage Registry are a good method to analyze and interpret data of this research field as they are able to reflect a large homogenous group of patient populations that resemble the common cartilage patient. Although randomized controlled trials (RCTs) remain the gold standard for evaluation in evidence-based medicine, there is often a shortcoming with this study design in terms of not being representative of patients of every day orthopaedic practice [31–33]. This could mean that only 4% of general cartilage patients undergoing surgical treatment are represented in common RCTs on cartilage repair [33]. This may be one of the reasons why only 3–6% of published articles in orthopaedics are RCTs [34].

On the other hand, this study is limited by issues commonly found in register studies. This especially includes selection bias [31, 35]. Another common problem that becomes evident when analyzing registry data is the unavoidable occurrence of missing data. In this study, preoperative data of 34 (27.0%) patients were missing, increasing to 48 missing patients (38.1%) for the 12-month follow-up data. However, reminder emails are sent out automatically by the German Cartilage Registry to minimize the number of non-responders.

## Conclusion

There seems to be an overall benefit of arthroscopic debridement for focal cartilage lesions with respect to the KOOS in the short-term follow-up. Isolated cartilage defects benefit from debridement irrespective of size. In patients with larger cartilage defects (> 2 cm^2^) and meniscal pathology expectation to improvement should be humbled. However, the benefit of effectively reducing the pain through arthroscopic debridement remains unclear. Further investigations regarding comparability and correlation of the KOOS-Pain and NRS for pain in a cartilage repair population need to be done.
