# Correction to: Is debridement beneficial for focal cartilage defects of the knee: data from the German Cartilage Registry (KnorpelRegister DGOU)

**DOI:** 10.1007/s00402-021-03905-0

**Published:** 2021-05-05

**Authors:** Manuel Weißenberger, Tizian Heinz, Sebastian P. Boelch, Philipp Niemeyer, Maximilian Rudert, Thomas Barthel, Stephan Reppenhagen

**Affiliations:** 1 Department of Orthopaedic Surgery, University of Wuerzburg, Koenig-Ludwig-Haus, Brettreichstr. 11, 97074 Wuerzburg, Germany; 2 OCM Clinic, Steinerstr. 6, 81369 Munich, Germany; 3 Department of Orthopaedics and Trauma Surgery, Freiburg University Hospital, Hugstetter Str. 55, 79106 Freiburg im Breisgau, Germany

## Correction to: Archives of Orthopaedic and Trauma Surgery (2020) 140:373–382 10.1007/s00402-020-03338-1

The article Is debridement beneficial for focal cartilage defects of the knee: data from the German Cartilage Registry (KnorpelRegister DGOU), written by Manuel Weißenberger, Tizian Heinz, Sebastian P. Boelch, Philipp Niemeyer, Maximilian Rudert, Thomas Barthel and Stephan Reppenhagen, was originally published Online First without Open Access. After publication in volume 140, issue 3, page 373–382 the author decided to opt for Open Choice and to make the article an Open Access publication. Therefore, the copyright of the article has been changed to © The Author(s) 2021 and this article is licensed under a Creative Commons Attribution 4.0 International License, which permits use, sharing, adaptation, distribution and reproduction in any medium or format, as long as you give appropriate credit to the original author(s) and the source, provide a link to the Creative Commons licence, and indicate if changes were made. The images or other third party material in this article are included in the article’s Creative Commons licence, unless indicated otherwise in a credit line to the material. If material is not included in the article’s Creative Commons licence and your intended use is not permitted by statutory regulation or exceeds the permitted use, you will need to obtain permission directly from the copyright holder. To view a copy of this licence, visit http://creativecommons.org/licenses/by/4.0/.

The original article has been corrected.

